# Antiobiotic use among Vietnamese hospitals: a multicenter cross-sectional study

**DOI:** 10.1186/1753-6561-5-S6-O42

**Published:** 2011-06-29

**Authors:** NV Hung, TA Thu, NQ Anh, S Salmon, D Pittet, ML McLaws

**Affiliations:** 1 Infection Control Department, Bach Mai Hospital, Ha Noi, Vietnam; 2 Bach Mai Hospital, Ha Noi, Vietnam; 3 School of Public Health & Community Medicine, University of New South Wales, Kensington, NSW, Australia; 4 Infection Control Programme, University of Geneva Hospitals and Faculty of Medicine, Geneva, Switzerland

## Introduction / objectives

Inappropriate antibiotic use is an important factor associated with antibiotic resistance and related medical costs. These outcomes impede the effectiveness of infection prevention control programmes. Our study is to determine the prevalence of antibiotic prescription and rationale for prescribed antibiotics to hospital inpatients in Vietnam.

## Methods

A one-day prevalence survey in 2008 was conducted in 36 hospitals representing three different hospital levels across Vietnam. Medical records of all inpatients were reviewed to collect demographics, number and antibiotic class, and indications for antibiotic prescription. Based on the guidelines of the Association for Professionals in Infection Control and Epidemiology, USA, reasons for antibiotic use were classified into (1) identified pathogen directed, (2) empirical, or (3) prophylactic.

## Results

The crude antibiotic use was 67.4% (3811/5654). Broad-spectrum antibiotics such as cephalosporins (70.3%), penicillins (21.6%), and aminoglycosides (18.9%) were most commonly used. Of antibiotic used patients, 54.7% were prescribed empirically and 30.8% were unclearly indicated. Risk factors independently associated with unclear antibiotic prescription were as follows: National level hospitals (adjusted odds ratio [aOR]: 2.2; 95% confidence interval [CI]: 1.7-2.9), provincial/regional hospitals (aOR: 1.3; CI: 1.1-1.6), obstetrics ward (aOR: 15.2; CI: 10.9-21.3), and surgical ward (aOR: 2.6; CI: 2.1-3.1).

## Conclusion

Suboptimal antibiotic prescription practices are common in our participating hospitals and emphasise the necessity for evidence-based guideline to be developed and implemented in Vietnamese hospitals.

## Disclosure of interest

None declared.

